# Important considerations when providing mental health first aid to Iraqi refugees in Australia: a Delphi study

**DOI:** 10.1186/s13033-016-0087-1

**Published:** 2016-09-01

**Authors:** Maria Gabriela Uribe Guajardo, Shameran Slewa-Younan, Yvonne Santalucia, Anthony Francis Jorm

**Affiliations:** 1 Centre for Health Research, School of Medicine, Western Sydney University, Sydney, Australia; 2 Health Promotion Service, Multicultural Health, South Western Sydney Local Health District, Sydney, Australia; 3 Centre for Mental Health, Melbourne School of Population and Global Health, University of Melbourne, Melbourne, Australia

**Keywords:** Mental health first aid, Iraqi refugees, Mental health problems, Delphi method

## Abstract

**Background:**

Refugees are one of the most vulnerable groups in Australian society, presenting high levels of exposure to traumatic events and consequently high levels of severe psychological distress. While there is a need for professional help, only a small percentage will receive appropriate care for their mental health concerns. This study aimed to determine cultural considerations required when providing mental health first aid to Iraqi refugees experiencing mental health problems or crises.

**Method:**

Using a Delphi method, 16 experts were presented with statements about possible culturally-appropriate first aid actions via questionnaires and were encouraged to suggest additional actions not covered by the questionnaire content. Statements were accepted for inclusion in a guideline if they were endorsed by ≥90 % of panellists as ‘*Essential*’ or ‘*Important*’.

**Results:**

From a total of 65 statements, 38 were endorsed (17 for cultural awareness, 12 for cross-cultural communication, 7 for stigma associated with mental health problems, and 2 for barriers to seeking professional help).

**Conclusion:**

Experts were able to reach consensus about how to provide culturally-appropriate first aid for mental health problems to Iraqi refugees, demonstrating the suitability of this methodology in developing cultural considerations guidelines. This specific refugee study provided potentially valuable cultural knowledge required to better equip members of the Australian public on how to respond to and assist Iraqi refugees experiencing mental health problems or crises.

**Electronic supplementary material:**

The online version of this article (doi:10.1186/s13033-016-0087-1) contains supplementary material, which is available to authorized users.

## Background

Australia is a multicultural country with almost a quarter (24.6 %) of its population born overseas and 43.1 % of people with at least one overseas-born parent [1]. Over the period 2003–2004 to 2012–2013, Australia resettled 146,321 refugees [2]. Most recently, the Australian Humanitarian Program 2013–2014 granted 13,768 visas to onshore and offshore visa applicants [3]. Currently, Iraq is one of the top source countries for refugee applications to Australia [3]. Further, in 2015, and in response to the conflicts in Syria and Iraq, the Australian Government announced an extra 12,000 humanitarian places available. People in these 12,000 places will be granted a permanent visa [4].

The presence of mental disorders amongst refugees has been well identified and explored for over 30 years [5–7]. Recent evidence has shown that prevalence of mental disorders and general psychological distress are both high in resettled Iraqi refugees [8, 9], with some experiencing severe psychological distress beyond the early resettlement period, even years after first arrival [9]. It has also been documented that while there is great need for specialised health care, only a small percentage of refugees will seek professional help [10], waiting until problems become critical before reaching specialist psychiatric and mental health services [11]. There may be several reasons why resettled Iraqi refugees do not seek help. Some of these may include stigma associated with mental health problems [12], inability to recognise mental disorders [13], lack of knowledge of treatment and intervention available in the host country [13] and migration challenges (e.g. cultural shock, lack of knowledge of the new country and its system, financial constraints, low language proficiency) [14].

Mental health first aid is *‘the help offered to a person developing a mental health problem, experiencing a worsening of an existing mental health problem or in a mental health crisis. The first aid is given until appropriate professional help is received or until the crisis resolves’* (p. 12) [21]. A Mental Health First Aid (MHFA) training program was introduced in Australia in 2001 with the goal of improving recognition of mental disorders, reducing stigma and promoting appropriate help-seeking, self-care and support from others in the community [15]. The Standard MHFA course is delivered over 12-h of training and teaches the application of a first aid action-plan to mental health problems [21]. It has since been evaluated in multiple studies in Australia and other countries, demonstrating its acceptability and effectiveness [16]. Remarkably, since 2001 MHFA has been disseminated rapidly and gained recognition, with over 20 countries adopting the MHFA course and adapting it to their specific needs [17–20].

To increase the evidence base of the MHFA program, a series of Delphi studies have been conducted to develop expert consensus guidelines on what constitutes appropriate mental health first aid for a range of mental health problems or crises, such as depression, anxiety, panic attacks, traumatic events, and suicidal behaviours [21]. This type of consensus study has been used widely in mental health research [22] and has also served in the development of additional guidelines on provision of culturally-specific mental health first aid for minorities in Australia [23, 24]. Minas and Jorm argue that *‘The development of a locally relevant evidence base is a valuable approach where other evidence is unavailable’* and that expert consensus methods are a way of achieving this (p. 2) [25]. The current study sought to develop guidelines on important additional considerations when providing mental health first aid to Iraqi refugees.

### Cultural factors and mental health in refugees

When assessing mental health needs of refugees, it is crucial to consider the complexity of the underlying phenomena and impact of various cultural perspectives, including Western approaches, in guiding understanding of mental illness. Over the past two decades, there has been a growing belief that the Western diagnostic and treatment systems for mental disorders may be insufficient or even in some cases inappropriate in cross-cultural settings [26]. Since then, it has become evident that gaining an appreciation of individuals’ understanding of mental health, their knowledge of treatments and willingness to access care for mental health concerns is essential when designing mental health services for the care of underrepresented minority groups (e.g. refugees or migrants) [27].

As such, in recent years, targeted research has emerged with the aim of exploring levels of mental health literacy amongst refugee groups in Australia [13, 28, 29]. These studies have reported lower recognition of mental health problems and differing beliefs regarding treatment approaches in refugee groups when compared to the Australian public [30]. Conclusions from these studies have highlighted the need for specific mental health education for refugees and those who care for them that embraces both Western mental health service approaches and traditional cultural and religious practices [13, 29].

Such culturally-responsive approaches have been used to help develop general health clinics [31], as well as other initiatives where asylum seekers and refugees have been trained to provide psycho-education and psychosocial support to fellow groups of refugees and asylum seekers [32].

This current study is expected to build cultural capacity at an Australian community level, by using a validated consensus method, which is deemed to be essential for effective cross-cultural interventions [23, 24, 33].

### The need for guidelines when assisting Iraqi refugees

Refugees have an elevated risk of mental disorders in the resettlement period as a consequence of significant personal disruption and experiences of torture, trauma, and loss that many have experienced [34], along with post-displacement challenges such as poor English language ability, unemployment, housing shortage and low educational opportunities [35].

In order to overcame the barriers in seeking professional help, researchers and clinicians are encouraged to develop alternative models of care to accommodate individuals’ cultural and linguistic backgrounds, including meanings of emotion, suffering, trauma and support in their original and host cultural contexts [11]. However, much more needs to be done to enhance mental health service utilisation amongst people from refugee backgrounds. One way forward is to develop community-based culturally-appropriate interventions which can tangibly benefit refugees by seeking to reduce their psychological distress at different points of their resettlement journey.

As such, this study sought to explore what culturally-appropriate mental health first aid strategies have been proposed for assisting Iraqi refugees in mental health crises or developing mental health problems and which of these are considered by experts in the field to be the most appropriate strategies for people who are assisting this group. The study used the Delphi consensus method by identifying and recruiting experts to rate the importance of action statements on how to provide culturally-appropriate mental health first aid to Iraqi refugees.

## Methods

### The Delphi method

First developed to examine the impact of technology on warfare in 1950s, it has been defined as a type of consensus method, often using a non-face-to-face technique, for structuring a group communication process, allowing a group of individuals to deal with a complex problem [36, 37]. It has been proven to be a feasible method when developing culturally-adapted interventions in mental health for minority and diverse groups [38, 39]. This method involves questionnaires being sent out online to a group of experts, where responses are anonymous. The Delphi technique involves a number of iterations before consensus is reached. Feedback from the expert group as a whole is given in order to assist panellists to assess their ratings against the group feedback.

The development of these guidelines involved 8 steps: (1) framing a research question, (2) formation of the panel (3) determining the expert panel size, (4) constructing the questionnaire, (5) information provided to panel members to aid their judgements, (6) administering the questionnaire, (7) analysing rounds and providing feedback to the panel and (8) reporting results.

### Research question

This study aimed to respond to the following questions: (1) *what culturally appropriate mental health first aid strategies have been proposed for assisting Iraqi refugees in mental health crises or in the development of mental health problems?* and (2) *which of these are considered by experts in the field to be the most appropriate strategies for assisting this group?*

### Formation of the panel

The expert panel was composed of professionals who meet the following selection criteria: qualified as a psychologist, social worker, psychiatrist, general practitioner or mental health professional; and have worked in refugee mental health part-time or full-time for at least 4 years and have experience working with Iraqi refugees. Potential participants were considered to have sufficient expertise if they have authored material in Iraqi refugee mental health and/or were known as experienced professionals through different networks, such refugee health networks in Australia, professional associations or community involvement.

Potential participants were identified and selected to participate in this project through their involvement with professional colleges and associations, universities and research centres, refugee health networks and refugee health services in New South Wales, South Australia, Australian Capital Territory and Victoria, Australia. Expert panellists were contacted via multiple sources. The principal researcher (MGU) advertised the study in various professional associations such as The Australian Psychological Society, The Australian Association of Social Workers, and The Royal Australian and New Zealand College of Psychiatrists. In addition, an email invitation was circulated to those involved with key government and non-government refugee health organisations and university centres. In the invitation to participate, professionals were asked to share the study invitation with colleagues who they felt would be appropriate panel members. This research was granted human research ethics approval by the Western Sydney University Human Research Ethics Committee (H11054). Consent and participation information sheet forms were sent by email or post to participants. Signed consent forms were collected by the principal researcher (MGU) before the study commencement. Participants were reimbursed for their time with a book voucher of $50.

### Determining expert panel size

A panel size of 23 has been found to yield stable results in a simulation study [40]. Our aim was for a minimum of 30 members in the panel, in order to allow for drop-outs. However, a total of 16 experts were recruited.

### Constructing the questionnaire

A systematic literature search was carried out to identify available information about how to provide culturally-appropriate mental health first aid to Iraqi refugees experiencing mental health problems or crises. This search focused in three main sources. A comprehensive search of key terms was carried out using Google search engines (http://www.google.com.ca, http://www.google.com.uk, http://www.google.com.au, http://www.google.com). The searches included various combinations of search terms, for example, *‘mental health in Iraqi refugees’* AND ‘*early mental health intervention for Iraqi refugees*’ AND *‘first aid for Iraqi refugees’* AND *‘cultural considerations for Iraqi refugee mental health’*. Lists of the first 50 websites for each set of terms were reviewed. Any links of interest appearing in the websites were followed, as appropriate. A second strategy was academic journal search using Medline, PsycINFO, CINAHL, PILOTS, Scopus and Cochrane databases to identify relevant published articles. A third strategy was searching for any printed books available in Australian-wide libraries using Trove Australia, Libraries Australia and Australian Libraries Getaway (ALG) search catalogues. Information from relevant mental health and refugee websites such as Transcultural Mental Health, Australian Psychological Society, Mental Health Australia and United Nations High Commissioner for Refugees (UNHCR) were also reviewed.

In order to develop the first round questionnaire, information obtained from the systematic literature review was divided into common sections or categories and were written as strategies or actions a first aider should follow. For example, in a report entitled *‘The Mental and Physical Health of Recent Iraqi refugees*’ it was stated that ‘*talking about mental health openly is often stigmatised by Iraqis’*. This statement was included in the first round questionnaire under the category ‘*stigma associated with mental health problems’* as a first aid action as follows: ‘*The first aider should be aware that talking about mental health problems openly is often stigmatised by the Iraqi community’*.

The process of phrasing and drafting statements into a questionnaire format involved a team of authors, who were experts in the Delphi method (AJ) or in transcultural mental health (MGU, SSY, YS). The team drafted the actions and attempted to remain as close to the original literature as possible. Statements were only modified in order to ensure format consistency or content comprehension. Several meetings were held to discuss the items before the final round 1 questionnaire was ready to be sent. In total, three questionnaires were created and presented to experts in a 3-round format.

### Information provided to experts to aid their responses

Questionnaires were accompanied by definitions of key concepts that were thought to be relevant for experts when making their ratings. These definitions included the role of a ‘first aider’, the ‘person’ defined as an ‘Iraqi refugee’ and ‘people’ defined as ‘Iraqi people in general’, as well as definitions of ‘mental Illness’, ‘cross-cultural communication’ and ‘cultural awareness’.

### Administering the questionnaire and analysing rounds

The majority of participants chose to complete the questionnaire (round 1) online and a link was sent hosted by http://www.SurveyMonkey.com to their nominated email addresses. A small number of experts preferred to complete a hard copy questionnaire. Experts responded to each statement by rating how essential the first aid action statements were to the development of guidelines on how to provide mental health first aid to Iraqi refugees experiencing mental health problems or crises. The questionnaire involved a 5-point Likert scale composed of ‘Essential’, ‘Important’, ‘Depends/don’t know’, ‘Unimportant’ and ‘Should not be included’ as response options.

Once the panel rating was completed, actions were categorized based of level of consensus following the procedure used in a previous Delphi study [23]:

1. If between 90 and 100 % of panel members rated a statement as either ‘Essential’ or ‘Important’, the statement was endorsed as a guideline.
2. If between 80 and 89 % of panel members rated a statement as either ‘Essential’ or ‘Important’, then the statement was entered into a second questionnaire to be rerated (second round).
3. If neither of the above conditions were met then the statement was excluded from the guidelines.

In round 1, in addition to rating statements, participants were encouraged to provide feedback on any ambiguity in the statements presented and suggest any new first aid strategies that were not included in the content of the questionnaire. Submitted comments were drafted into statements and then presented to the working group, who tried to ensure comprehensibility and consistency. Those statements that were assessed by the working group to be original were included as new actions in a second round questionnaire for experts to rate.

Once categorisation was completed, participants were sent a report that included endorsed, rejected and new statements to be rated along with those statements that needed to be re-rated in the next round questionnaire. The statements to be re-rated were presented with the group percentages for each possible rating, and also with the individual’s response for the expert to compare their own rating to the group response. By providing this report to the experts, researchers aimed to aid participants’ re-rating.

In round 2, the same criteria were followed for endorsing, excluding and re-rating statements. However, those statements that failed to meet the criteria for endorsement in the second round were then excluded from the guidelines. Only those new statements that were introduced in round 2, and afterward fell into the re-rate category, were in a third and final round.

## Results

Sixteen panel members were recruited (7 male, 9 female, mean age = 44.37; SD = 12.46) with 100 % retention across the three rounds. The majority of recruited participants were based in Australia, with only one expert based in the Netherlands. Eleven participants were from New South Wales, three from South Australia and one from Australian Capital Territory.

The majority worked in the private sector and years of experience ranged from 4 to 40 (mean = 16.55; SD = 11.44) with more than 40 % having 16 or more years of experience.

Participants’ professions varied and included: consultant psychiatrists (n = 2), psychologists (n = 4), general practitioners (n = 4), medical director (n = 1), case managers/social workers (n = 3), mental health project officer (n = 1) and occupational therapist (n = 1). It is important to note that some of the participants held both professional and academic appointments (n = 3). Most of participants had attained a post-graduate degree (n = 11).

### Endorsed statements

**Fig. 1:**
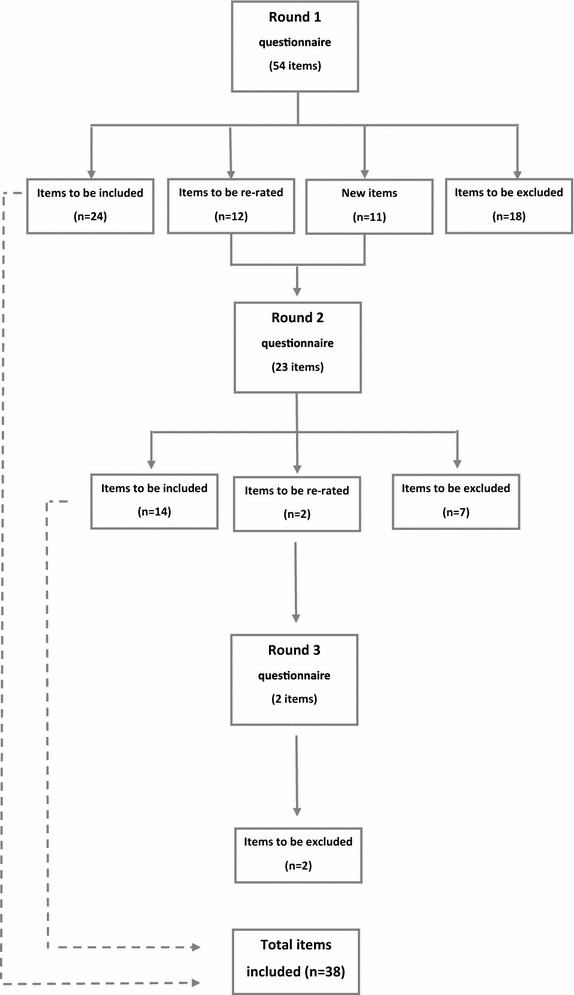
The Delphi process in three consecutive rounds

Of the 65 statements presented to the experts, including those statements that were drafted from experts’ feedback, 38 were endorsed as either *essential* or *important* to the development of guidelines on providing mental health first aid to Iraqi refugees (Fig. 1). A list of all endorsed statements can be found in Additional file 1. Endorsed items were categorised in four main topics as follows: cultural awareness, cross-cultural communication, stigma associated with mental health problems and barriers to seeking professional help (Table 1). The vast majority of endorsed statements fitted within the cultural awareness category due to the relatively larger literature in this area compared to the other categories. In terms of round inclusion, the majority of the statements were endorsed in round 1 (n = 24), followed by round 2 (n = 14). There were no statements endorsed in round 3.

### Rejected statements

A total of 27 statements were excluded from the guidelines as it failed to reach the 90 % level as *‘Important’* or *‘Essential’* (Fig. 1). A list of rejected items can be found in Additional file 2. The majority of rejected statements were categorised under *‘Cultural awareness’*, consistent with the pattern reported for endorsed statements (Table 1). Again, the majority of the statements were rejected in round 1.

**Table 1:** Summary of endorsed and rejected statements by round and category

Round	Items							
	Endorsed				Rejected			
	Cultural awareness	Cross-cultural Communication	Stigma	Barriers to seeking help	Cultural awareness	Cross-cultural communication	Stigma	Barriers to seeking help
1	11	9	3	1	6	3	3	6
2	6	3	4	1	4	–	2	1
3	–	–	–	–	–	1	1	–

### New statements

In round 1, researchers encouraged panel members to provide feedback or any strategies they thought would be useful when providing mental health first aid to Iraqi refugees. A total of 11 new strategies based on the panel’s feedback were incorporated in round 2 (Fig. 1). Six statements were endorsed as either *essential* or *important* by the panel. These statements were endorsed in round 2, with no new strategies endorsed in round 3.

## Discussion

The current study sought to explore what experts in refugee mental health thought would be culturally-appropriate mental health first aid strategies for assisting Iraqi refugees in mental health crises or developing mental health problems. Experts were able to reach consensus on the importance of a set of culturally-specific guidelines on how to provide mental health first aid to Iraqi refugees with high levels of distress.

The first category that emerged was ‘cultural awareness’. Research has demonstrated that understanding cultural norms, values and beliefs of a person from a different cultural background is essential when trying to help individuals in cross-cultural contexts [23, 24, 34]. Thus, this category represents the importance of cultural awareness when providing mental health first aid to Iraqi refugees. This awareness can aid the identification of presenting problems (mental health symptoms) and provision of appropriate assistance, going beyond standard mental health classifications. Specifically, members of the public who wish to assist resettled Iraqi refugees should be aware of wars and mass persecution that Iraq has endured for almost three decades, including internal and overseas displacement of its population. It is also recommended that aiders should have awareness of the diversity of ethnicities and religions in Iraq and among those who have being granted permanent resettlement in Australia. Further, aiders must also understand the impact of culture on attitudes, behaviours and beliefs associated with mental health problems and mental health risk factors in the Iraqi community. These strategies reflect the importance of complexity of Iraq’s cultural, ethnic and religious homogeneity and how such differences can play a role when assisting individuals from Iraq.

It also became evident, as noted previously [23, 24, 34], that applying ‘cross-cultural communication’ strategies while communicating with individuals from a different culture is essential. According to the results of this study, aiders should be encouraged to communicate in a culturally sensitive and respectful way, being mindful of the limited English skills refugees may have, which can increase the potential for misunderstandings. There is no doubt that communicating with individuals with limited English skills or from culturally diverse backgrounds can be challenging, and that is why aiders are encouraged to promote the use of a professional interpreter when one is needed. Aiders should be aware of the environment and surroundings when trying to help refugees, avoiding places that might trigger traumatic memories of when they fled home, including their refugee journey (internally displaced or displaced in neighbouring countries) before arriving to Australia. Observing these recommended strategies is essential for anyone wanting to offer effective initial help to someone from an Iraqi refugee background. By undertaking such strategies, communication is enhanced and potential misunderstandings are avoided, allowing first aiders to effectively approach, assist and assess for mental health crises, listen, give information and encourage professional help and other supports, as recommended in the action plan taught in MHFA courses [21].

A third category that emerged was ‘stigma associated with mental health problems’ in the Iraqi community. Negative attitudes towards mental health problems have been reported as one of the major reasons preventing people from seeking professional help in Australia [30]. In line with this evidence, stigma associated with mental health problems seems to be a rule in many countries and across different groups [41–43]. Specifically, Bolton [12] reported that the stigma associated with mental disorders in Iraq seems to be greater than in other parts of the world. According to Bolton, social distance and the stigma of mental illness extends to the patients’ families, with feelings of shame as a common feature [12]. The current study identified that Iraqi refugees can be considered as ‘crazy’ by their community if they speak out about their mental health concerns. Aiders should be mindful that Iraqis may keep their mental health problems as secret and may not trust authorities such as health professionals, and that this can act as a barrier.

The last set of recommendations were categorised as ‘barriers to seeking professional help’. Professional help-seeking is defined as the assistance from professionals who have a legitimate and recognised professional role in providing relevant advice, support and/or treatment. These include specialist and generalist health care providers [44]. Research has indicated that only a small number of refugees will seek professional help for mental health problems, with non-professional help-seeking being the preferred source of help for refugees during a mental health crisis [45]. Hence it is essential for members of the public wanting to assist Iraqi refugees to be aware that only a limited number of refugees will seek professional help. There is also a tendency for men not to seek help for fear of appearing weak when they are expected to be strong and support their families. In addition, traditional beliefs and causes of mental health problems may also prevent Iraqis from seeking professional help. It is important to understand the great disruption and the new identities that some refugees are forced to assume. First aiders needed to be mindful that Iraq is a very patriarchal society where traditional beliefs and gender roles are strongly adhered to and that such beliefs can impact professional help-seeking from individuals.

Limitations in this study must be noted. This study utilised a volunteer process and experts were recruited through their association with key non-government and government organisations. This method may have failed to capture experts not directly connected to such organisations, but who may have a more grass roots connection in assisting Iraqi refugees. In addition, the sample size of this study was relatively small with only 16 panel members recruited. This number, while smaller than the optimal panel size of 23 demonstrated to yield stable results in a simulation study [40], is likely to be a reflection of the relatively limited number of experts available on Iraqi mental health.

Future directions should include ways to engage and recruit mental health professionals in order to ensure a larger sample size, as well as engaging with carers and consumers from refugee backgrounds to be part of a multiple ‘expert panels’ study. However, this may impose a new challenge due to the difficulty of recruiting samples of individuals with a refugee background [46].

Strength of this research includes that this is the first study aiming to establish culturally-appropriate evidence-based actions to assist individuals from an Iraqi refugee background. By building on the highly-successful standard MHFA training, our guidelines will inform tailored education on how to help an Iraqi refugee presenting with common mental health problems such as depression and posttraumatic stress disorder among others. Additionally, while this study focussed on a specific refugee group, this method appears well suited in developing other cultural specific guidelines. Future directions will include the delivery and evaluation of this tailored educational program, which is currently underway and involves recruiting and training community-based workers assisting resettling Iraqi refugees in Western Sydney, Australia. It is anticipated that this tailored training will form the first line of a two-pronged approach seeking to improve the mental health outcomes of Iraqi refugees, with the second being mental promotion and educational strategies targeted towards the Iraqi refugees themselves.

## Conclusion

In summary, our Delphi study is the first conducted with a specific refugee group and demonstrated the need for first aiders to have specific knowledge that can be categorised in four broad areas; (1) knowledge of the Iraqi culture and community in Australia; (2) how to communicate effectively with individuals from an Iraqi background (3) traditional beliefs about causes and negative attitudes towards mental health problems held by this group; and (4) common barriers to seeking professional help that can be present in resettled Iraqi refugees. These guidelines are relevant and needed, especially given that Iraq is one of the top-source countries of refugee applications to Australia. These guidelines represent the *first step on the road* in order to improve professional help-seeking in Iraqi refugees with mental health problems.

## Additional files

10.1186/s13033-016-0087-1 Endorsed items by category.
10.1186/s13033-016-0087-1 Rejected items by category.

## Abbreviations

MHFA: Mental Health First Aid
ALG: Australian Libraries Getaway
UNHCR: United Nations High Commissioner for Refugees
