# Supplementary material for: Important considerations when providing mental health first aid to Iraqi refugees in Australia: a Delphi study
Source: Int J Ment Health Syst. 2016 Sep 1;10(1):54. doi: 10.1186/s13033-016-0087-1 (PMC5009547; doi:10.1186/s13033-016-0087-1)
Supplement: Supplementary file 1 — 10.1186/s13033-016-0087-1 Endorsed items by category. [file 13033_2016_87_MOESM1_ESM.docx]

Additional file 1 **– Endorsed items by category**

| **Round** | **Endorsed statements by category** |
| --- | --- |
|  | *Cultural awareness (n=17)* |
| 1 | The first aider should be aware that, compared to other refugee groups, the person may have poorer general health and greater exposure to war-related mental and physical trauma. |
| 1 | The first aider should be aware that, compared to the general Australian population, the person may have poorer mental health. |
| 1 | The first aider should be aware that after the person comes to Australia, they can experience major disruption in their gender role which can be an additional source of distress. |
| 1 | The first aider should be aware of cultural beliefs about the causes of mental health problems that the person may hold. |
| 1 | The first aider should be aware of the risk factors for mental health problems in Iraqi refugees. |
| 1 | The first aider should be aware that ongoing conflicts in Iraq may trigger or worsen mental health problems in the person. |
| 1 | The first aider should be careful to respect the cultural traditions of the person, and not to dismiss or trivialise them. |
| 1 | The first aider should avoid making generalisations about the person based on their Iraqi background, as there is significant ethnic and religious diversity in this group. |
| 1 | The first aider should avoid making assumptions or generalisations based on the person's background and should ask the person directly. |
| 1 | The first aider should beware of attributing too much to culture and ethnicity, particularly as there is a range of factors affecting refugees' mental health problems (e.g. trauma and torture, experiences in their country of origin, settlement issues). |
| 1 | The first aider should be aware of the potential influence that their own culture, values, expectations and attitudes can have on the help they give to the person. For example, the first aider's confidence in Western approaches to health may lead them to overlook or dismiss alternative health beliefs that may be held by Iraqi people. |
| 2 | The first aider should be aware that there might be a tendency for Iraqi refugees with mental health problems to present somatic complaints rather than psychological ones. |
| 2 | The first aider should be aware that the person is unlikely to have knowledge of psychotherapy as a treatment option for mental health problems. |
| 2 | The first aider should be aware that the person's outlook and well-being may change as they encounter different stages of resettlement (e.g. honeymoon period immediately following arrival giving way to increased stress in response to everyday difficulties). |
| 2 | The first aider should be aware that a person may use alternative words to express their distress (e.g. using the word 'nerves' rather 'anxiety') (New in R2). |
| 2 | The first aider should be aware that the person may feel fear of authorities, including health professionals, which may result in filtering information when discussing their mental health problems (New in R2). |
| 2 | The first aider should know the historical context of the conflicts and wars that Iraq has been engaged in over the past 30 years, which may affect the person's mental health and help-seeking behaviour (New in  R2). |
|  | *Cross-cultural communication (n=12)* |
| 1 | The first aider should be aware that a person who has only recently arrived in Australia may have rudimentary English comprehension and Australian cultural insight, which may increase the potential for misunderstanding. |
| 1 | The first aider should be aware that politeness may lead the person to indicate that they have understood when this is not so. |
| 1 | The first aider should offer a professional interpreter to the person, where one is needed. |
| 1 | The first aider should explain to the person the role of any interpreter and their obligations to keep information confidential. |
| 1 | The first aider should be aware of the telephone interpreting services, which are nationally available. |
| 1 | The first aider should be aware of the importance of using a professional interpreter, in order to avoid ethical and safety issues associated with using a family member, friend, or bilingual employee. |
| 1 | The first aider should encourage the person to use interpreting services when consulting with health providers and other social services. |
| 1 | If an interpreter is required, the first aider should establish the person’s preferred language, ethnicity and gender of the interpreter. |
| 1 | If an interpreter is required, the first aider should allow for additional extra time involved in communicating through an interpreter. |
| 2 | The first aider should think about their surroundings and avoid having conversations in places that may trigger flashbacks and fearful reactions in the person (e.g. rooms with closed-in spaces or barred windows). |
| 2 | The first aider should avoid using a raised voice when communicating with a person with limited English comprehension, as this will not enhance communication. |
| 2 | If the person does not appear to understand, the first aider should rephrase their statement in simpler words, avoiding jargon (New in R2). |
|  | *Stigma associated with mental health problems items (n=7)* |
| 1 | The first aider should know that a person with mental health problems might feel ashamed if they seek treatment. |
| 1 | The first aider should be aware that a person with mental health problems may not seek professional help for fear of prejudice from their community. |
| 1 | The first aider should be aware that mental health problems are often considered as a weakness within the Iraqi refugee community. |
| 2 | The first aider should know that a person with mental health problems can be considered 'insane' or' crazy' by members of their community. |
| 2 | The first aider should be aware that talking about mental health problems openly is often stigmatised by this culture. |
| 2 | The first aider should avoid making assumptions or generalisations about the person's beliefs about mental health problems and the associated stigma (New in R2). |
| 2 | The first aider should know the person may keep mental health problems a secret, especially when seeing a health professional (New in R2). |
|  | *Barriers to seeking professional help items (n=2)* |
| 1 | The first aider should be aware that men might not seek professional help because they do not want to appear weak because they are required to be strong in order to support their families. |
| 2 | The first aider should be aware that the common traditional explanations for mental health problems may act as barriers to seeking professional help within the Australian health system |
|  |  |
