# Supplementary material for: Important considerations when providing mental health first aid to Iraqi refugees in Australia: a Delphi study
Source: Int J Ment Health Syst. 2016 Sep 1;10(1):54. doi: 10.1186/s13033-016-0087-1 (PMC5009547; doi:10.1186/s13033-016-0087-1)
Supplement: Supplementary file 2 — 10.1186/s13033-016-0087-1 Rejected items by category. [file 13033_2016_87_MOESM2_ESM.docx]

Additional file 2 – **Rejected items by category**

| **Round** | **Rejected statements by category** |
| --- | --- |
|  | *Cultural awareness (n=10)* |
| 1 | The first aider should be aware that the person often faces lack of local support or trusting relationships with others in Australia. |
| 1 | The first aider should be aware that superstition and pagan beliefs around mental health problems are mainly present amongst people with less education or from a rural background. |
| 1 | The first aider should be aware that people from more urban backgrounds are more likely to have concepts of illness that approximate those of the West. |
| 1 | The first aider should be aware that, under Saddam's regime, there was a belief that mental health problems could not happen to people who were faithful Muslims. |
| 1 | The first aider should be aware that the person may want to involve family members in any discussion about seeking professional help for their mental health problems. |
| 1 | The first aider should be aware that some religious practices (e.g. reading religious texts or praying) can be helpful when dealing with mental health problems. |
| 2 | The first aider should be aware that, compared to the general Australian population, the person may have poorer general health. |
| 2 | The first aider should be aware that people from Iraq are reluctant to engage with treatment for mental health problems. |
| 2 | The first aider should be aware that cultural explanations held by the person for mental health problems may be attributed to external causes (e.g. divine or spiritual causes). |
| 2 | The first aider should be aware that having an exhaustive knowledge about specific cultural beliefs may not be possible (New in R2). |
|  | *Cross-cultural communication (n=4)* |
| 1 | The first aider should be aware that Iraqi refugees often face trouble communicating and expressing feelings. |
| 1 | The first aider should consider allowing trusted persons to be present when discussing the person's mental health problems. |
| 1 | If the person wants to recount their refugee journey, the first aider should listen without interruption. |
| 3 | When using an interpreter, the first aider should set the ground rules of how the interpreting will be done prior to the discussion with the person (e.g. simultaneous versus consecutive interpreting and that every word needs to be translated versus paraphrasing) (New in R2). |
|  | *Stigma associated with mental health problems items (n=6)* |
| 1 | The first aider should be aware that stigma associated with mental health problems in Iraq seems to be greater than in other parts of the world. |
| 1 | The first aider should be aware that the stigma of mental illness extends to families, with many Iraqis reluctant to marry into families in which one or more members are mentally ill. |
| 1 | The first aider should be aware that many Iraqis believe that people suffering from mental health problems are to blame for their condition. |
| 2 | The first aider should be aware that some Iraqis believe that mental health problems can come as a punishment due to not fulfilling their religious duties (New in R2). |
| 2 | In order to overcome the stigma of professional help-seeking, the first aider should suggest that the person seeks an assessment from a health professional rather than help for mental health problems (New in R2) |
| 3 | The first aider should be aware that the person may not seek professional help unless they are encouraged to do by Government organisations (e.g. Centrelink) (New in R2). |
|  | *Barriers to seeking professional help items (n=7)* |
| 1 | The first aider should be aware that coming from a collective society may act as a barrier in seeking professional help within the Australian health system, because the individual's need may be secondary to the needs of the family unit. |
| 1 | The first aider should be aware that women may be stopped from seeking professional help by their male relatives. |
| 1 | The first aider should know that a person with mental health problems may prefer to seek help from their family rather than seeking professional help. |
| 1 | The first aider should know that a person with mental health problems may prefer to seek help from their religious leaders rather than seeking professional help. |
| 1 | The first aider should understand that even when a person wants treatment, they may be unable to seek it without family consent, particularly women who may require a male chaperone to travel outside home. |
| 1 | The first aider should know that a person with mental health problems may prefer to seek help from traditional healers rather than seeking professional help. |
| 2 | The first aider should be aware that the person may prefer to seek guidance from someone of the same gender. |
|  |  |
